# Changes in Microbiome Activity and Sporadic Viral Infection Help Explain Observed Variability in Microcosm Studies

**DOI:** 10.3389/fmicb.2022.809989

**Published:** 2022-03-16

**Authors:** Helena L. Pound, Robbie M. Martin, Brittany N. Zepernick, Courtney J. Christopher, Sara M. Howard, Hector F. Castro, Shawn R. Campagna, Gregory L. Boyer, George S. Bullerjahn, Justin D. Chaffin, Steven W. Wilhelm

**Affiliations:** ^1^Department of Microbiology, The University of Tennessee, Knoxville, TN, United States; ^2^Biological and Small Molecule Mass Spectrometry Core, The University of Tennessee, Knoxville, TN, United States; ^3^Department of Chemistry, State University of New York, College of Environmental Science and Forestry, Syracuse, NY, United States; ^4^Department of Biological Sciences, Bowling Green State University, Bowling Green, OH, United States; ^5^Stone Laboratory and Ohio Sea Grant, The Ohio State University, Put-In-Bay, OH, United States

**Keywords:** transcriptomics, metabolomics, cyanobacterial blooms, fresh waters, nutrients, *Microcystis*

## Abstract

The environmental conditions experienced by microbial communities are rarely fully simulated in the laboratory. Researchers use experimental containers (“bottles”), where natural samples can be manipulated and evaluated. However, container-based methods are subject to “bottle effects”: changes that occur when enclosing the plankton community that are often times unexplained by standard measures like pigment and nutrient concentrations. We noted variability in a short-term, nutrient amendment experiment during a 2019 Lake Erie, *Microcystis* spp. bloom. We observed changes in heterotrophic bacteria activity (transcription) on a time-frame consistent with a response to experimental changes in nutrient availability, demonstrating how the often overlooked microbiome of cyanobacterial blooms can be altered. Samples processed at the time of collection (T0) contained abundant transcripts from Bacteroidetes, which reduced in abundance during incubation in all bottles, including controls. Significant biological variability in the expression of *Microcystis*-infecting phage was observed between replicates, with phosphate-amended treatments showing a 10-fold variation. The expression patterns of *Microcystis*-infecting phage were significantly correlated with ∼35% of *Microcystis*-specific functional genes and ∼45% of the cellular-metabolites measured across the entire microbial community, suggesting phage activity not only influenced *Microcystis* dynamics, but the biochemistry of the microbiome. Our observations demonstrate how natural heterogeneity among replicates can be harnessed to provide further insight on virus and host ecology.

## Introduction

Research in environmental microbial ecology is often driven by observations and survey-style data where samples are gathered from natural systems during transects or time series to provide important insight into the spatial and temporal diversity and function of microbial communities (e.g., Venter et al., 2004). Yet, in many cases, the ability to test hypotheses (Popper, 1959) requires experimental manipulation of the entire community under natural conditions. To do this, aquatic researchers often employ “bottle experiments” where water samples are collected and distributed across a series of incubation vessels. Bottle experiments have been used for many decades (Schelske, 1984) and are advantageous because the duration of the experiments is similar to the generation time of the organisms being studied (Sterner, 2008). Incubation vessels, or bottles, are manipulated experimentally by introducing biological, chemical, or physical variation to a subset of the bottles that can be compared to unamended “control” bottles. Many experiments of this nature have been completed and used to document processes that range from the role of iron (Fe) as a growth-limiting element in marine systems (Martin and Fitzwater, 1988; Hutchins et al., 1999) to the rate at which virus populations are actively produced within microbial communities (Wilhelm et al., 2002). In fresh waters, these types of experiments have been used extensively to resolve the effects of nutrient additions on planktonic communities (Wilhelm et al., 2003; DeBruyn et al., 2004; Xu et al., 2010). Despite their detractors (Carpenter et al., 1995; Schindler, 1998), incubation experiments remain fundamental to the aquatic microbial ecologists’ toolset.

The process of executing a well-designed experiment in an enclosure, such as the common bottle-based experiments, is challenging and requires that attention be paid to spatial and temporal scales that could influence the outcome of the experiment and the interpretation of the results. The simple act of placing water containing a natural phytoplankton community into an enclosed bottle can result in differences which are often considered to be “environmental uncertainty” or “bottle effects” (Stewart et al., 2012). Variability due to the limited volume of a container has been well documented: the simple act of controlling temperature and light across different container sizes is challenging (Spivak et al., 2011). Enclosed bottle experiments allow for independent successional patterns and levels of heterogeneity, even amongst replicate bottles of the same treatment. Additionally, unlike natural systems, regenerative processes, inputs, and sinks for nutrients and dissolved gases are often artificially restricted in a microcosm (Zobell and Anderson, 1936), with cascading nutrient limitations as a result (Davidson and Howarth, 2007). The presence of a few large grazers in bottle experiments can also introduce noise into the data (e.g., Vanni and Temte, 1990; Urrutia-Cordero et al., 2015). Hypothetically, when water containing natural plankton communities are incubated in several bottles under identical conditions, the response metric measured at the end of the experiment should be consistent among replicates; however, variation is always observed, but not always explained (*e.g.*, see references above).

While many researchers focus only on phytoplankton responses in bottle experiments, it is well-established that heterotrophic bacteria in the phycosphere profoundly impact phytoplankton (Cook et al., 2020; Jankowiak and Gobler, 2020). Understanding the responses and variabilities associated with heterotrophic bacteria placed in a bottle may help explain variation, because slight changes to the microbiome can lead to notable differences in microbial diversity and function over time: even when an experiment starts from a consistent microbiome, it can quickly change identity and function (Pound et al., 2021a).

Perhaps more challenging is resolving the influence of viruses, which are ubiquitous in natural systems and key drivers of processes at the biogeochemical, population, and ecosystem levels (Wilhelm and Suttle, 1999; Breitbart, 2012; Sullivan et al., 2017). Unlike copepods and other grazers, viruses are often “invisible” to researchers (Roux et al., 2015) and thus are overlooked as possible drivers of biological variability (Pound et al., 2020). Yet when viruses in a system actively infect a population, the resulting cell lysis (Fuhrman, 1999; Wilhelm and Suttle, 1999) and/or viral-induced reshaping of gene expression can have strong community scale effects (Lindell et al., 2005; Forterre, 2011). Viruses have been shown to be constant companions in *Microcystis*-dominated communities (Yoshida M. et al., 2008; Yoshida et al., 2010) and to play potentially important roles in system ecology (Steffen et al., 2017).

To investigate the effects of short-term nutrient pulses during *Microcystis* blooms in the Laurentian Great Lakes, we conducted *in situ* bottle incubations comparing unamended controls to nutrient amendments over 48 h. We examined the transcriptome and metabolome of the entire community in independent replicates with the aim of identifying community responses to episodic nutrient introductions. During our analyses, we noted variability between treatments as well as between replicates within treatments. We examined this variation in an attempt to categorize outlier observations as due to either “unexplained random error” or “biological activity.” Our observations demonstrated not only how nutrient additions influenced transcriptional and metabolic landscapes of a toxic cyanobacterial bloom, but also how the confluence of experimental effects and natural variation can be resolved, including the variation arising from the influence of viruses on microbial communities.

## Materials and Methods

### Experimental Setup for Incubations and *in situ* Measurements

Lake water was collected in 20 L carboys from the surface of a *Microcystis* spp.- dominated bloom in Lake Erie, (United States) on July 21st, 2019 (41° 44.946′ N 83° 06.448′ W). Water column physiochemistry was recorded prior to sampling using an EXO multiparameter sonde (YSI xylem). Homogenized water was aliquoted into 18 × 1.2 L polycarbonate bottles and subject to four nutrient addition treatments in biological triplicate: 1 μM phosphate as KH_2_PO_4_:K_2_HPO_4_ (Belisle et al., 2016), and 180 μM nitrogen as either stable-isotope-labeled nitrate (^15^NO_3^–_), stable-isotope-labeled ammonia (^15^NH_4^+_), or stable-isotope dual-labeled urea (^13^CH_4_^15^N_2_O). Non-replicated bottles of non-labeled nitrate, ammonia, and urea additions were also included to act as standards for metabolome normalization (see metabolite methods below). Six bottles were included as controls with no nutrient additions, three of which were sampled immediately and considered time zero samples. The remaining three control bottles and all nutrient-amended bottles were incubated *in situ* in Lake Erie off docks at Stone Laboratory (41° 39.467′ N 82° 49.600′ W) for 48 h.

Samples were collected at 48 h for metabolomics analysis and RNA sequencing, as well as to measure pH, nutrients in the dissolved fraction, chlorophyll *a*, and toxins. Total sample collection time took ∼ 90–120 min. Approximately 180 mL of water from each bottle was collected on 0.2-μm GTTP filters (EMD Millipore Corporation, Burlington, MA, United States), stored in 2-mL cryovials and flash-frozen in liquid nitrogen for metabolomics analysis (see below). Approximately 150 mL of water from each bottle was filtered through a 0.2-μm nominal pore-size Sterivex™ filter (EMD Millipore Corporation, Burlington, MA, United States) and flash-frozen in liquid nitrogen for subsequent RNA extraction and sequencing (see RNA extraction methods below). pH was measured *via* immediate readings of 15 mL subsamples using a pH probe (Mettler Toledo Seven Compact™ pH/Ion meter S220, fitted with a Mettler InLab Expert Pro-ISM electrode with a temperature range of up to 100°C). Approximately 50 mL of 0.2-μm-filtered water was captured from the Sterivex filtrate for dissolved nutrient analysis (nitrate, ammonia, and phosphate) and stored at 4°C until processing on a SEAL Analytical (Mequon, WI, United States) QuAAtro 5-Channel continuous segmented flow auto-analyzer (Chaffin et al., 2019). Approximately 100 mL of water was filtered onto 0.2-μm nominal pore-size 47-mm diameter polycarbonate filters, and chlorophyll *a* extracted in 90% acetone for 24 h at 4°C prior to measurement on a Turner Designs 10-AU Field Fluorometer (Welschmeyer, 1994). Intracellular microcystin concentrations were determined using HPLC coupled with single quadrupole mass spectroscopy and photodiode array spectroscopy as described by Tang et al. (2018) and Boyer (2020). Standards (microcystin-RR, -LR and -LF) were analyzed at the beginning and end of each run to ensure that the retention times did not drift, and individual toxin congeners (RR, YR, and LR) were identified based on retention time, characteristic absorbance spectrum in the photodiode array detector, and characteristic molecular ions.

### RNA Extraction, Processing, and Sequencing

RNA extraction, sequencing, and quality control details can be found in previous publications (Tang et al., 2018; Pound et al., 2020). A step-by-step protocol describing the acid-phenol-based RNA extraction method with an added DNase treatment to remove any residual DNA is available (Pound and Wilhelm, 2020a). RNA quality was checked using a NanoDrop ND-1000 spectrophotometer (Thermo Fisher Scientific, Walthan, MA, United States) and quantified using the Qubit hsRNA assay (Invitrogen, Waltham, MA, United States). Extracted RNA was processed using an Illumina ^®^ Stranded Total RNA Prep, Ligation with Ribo-Zero Plus (for 96 Samples) and then 50-million 100-bp paired-end reads were generated on the Illumina NovaSeq platform at Hudson Alpha Discovery Life Sciences (Huntsville, AL, United States). Raw reads are available on the NCBI SRA database under BioProject number PRJNA737197. Sequence processing and assembly details can be found in an online, step-by-step protocol (Pound and Wilhelm, 2021b). Briefly, sequences were quality controlled and trimmed in the CLC Genomics workbench version 20.0.4 (Qiagen, Germantown, MD, United States). Lingering ribosomal rRNA reads were removed *in silico* using SortMeRNA version 4 (Kopylova et al., 2012). The non-ribosomal trimmed reads from all samples were jointly assembled in MegaHit version 1.2.9 (Li et al., 2015). As previously described (Pound et al., 2020), this was done to reduce redundancy among identical sequences in various samples.

### Characterization of Microbiome in Metatranscriptome Assemblies

Gene coding sequences were predicted from nucleotide and translated amino acid sequences using MetaGeneMark version 3.25 (Besemer and Borodovsky, 1999; Zhu et al., 2010). Amino acid sequences were functionally and taxonomically annotated *via* KEGG orthology using the prokaryote + eukaryote + virus database in GhostKOALA (Kanehisa et al., 2016; Pound et al., 2021b). KEGG orthology numbers (KOs) were assigned to predicted coding sequences and transcript expression was estimated by recruiting trimmed reads to the coding sequences using a 90% similarity fraction over a 90% length fraction in CLC Genomic Workbench. Reads that could be recruited to more than one contig with equal identity were randomly assigned. The KEGG orthology annotations provided by GhostKOALA allow for estimates of individual taxa and/or individual annotated genes or functional groups. DNA-dependent RNA polymerase (*rpoB*) expression was used to quantify active transcription as a proxy for organism abundance. Differential expression analysis of *Microcystis-*specific functional genes annotated with KOs was performed using DESeq2 (Love et al., 2014).

### Characterization of Virus Signatures in Metatranscriptome Assembly

Identification and transcript quantification of viruses of interest was performed using a BLASTx method previously described (Pound and Wilhelm, 2019, 2020b). Phage protein databases were established for the following genes of interest: terminase, major capsid protein, and tail sheath with all reference sequences downloaded from UniProt KB database version 2020_03 (see data availability). The assembled contigs from all samples were then queried using the command-line BLASTx against each database specific to a gene of interest, retaining sequences with a minimum contig length of 300 bp and an *e*-value < 1 × 10^–20^ (Camacho et al., 2009). Contigs with BLASTx hits were considered “candidates” and only the aligned portion of the gene sequence was used for subsequent expression analyses (Pound and Wilhelm, 2019). Non-ribosomal trimmed reads from each sample were recruited to aligned portion of each candidate contig in CLC Genomics workbench version 20.0.4 (Qiagen) using a 90% length fraction and 90% identity (Pound and Wilhelm, 2020b). Non-specific recruitments (reads that could be recruited to more than one contig with equal identity) were randomly assigned, to ensure all reads were quantified. Phylogenetic trees were established with maximum likelihood phylogenies based on reference proteins from isolated organisms using PhyML version 3.0 (Guindon et al., 2010). To determine taxonomy, candidate sequences were placed on reference phylogenetic trees using the pplacer algorithm (Matsen et al., 2010). Transcript expression for the entire *Microcystis* phage genome was estimated by recruiting reads to the *Microcystis* phage Ma-LMM01 genome (Accession AB231700.1) using a 90% length fraction and 90% identity (Yoshida T. et al., 2008; Pound and Wilhelm, 2020b).

### Metabolite Extraction and Assessment

Samples for metabolomics analysis were processed at the Biological and Small Molecule Mass Spectrometry Core (BSMMSC), University of Tennessee, Knoxville, TN (RRID: SCR_021368) in a manner similar to Krausfeldt et al. (2019). In brief, filters with biomass were exposed to pre-chilled extraction solvent (1.3 mL of 40:40:20 HPLC grade acetonitrile/methanol/water with 0.1 M formic acid) at −20°C for 20 min before water soluble metabolites were extracted from filters at 4°C. Extracted metabolites were dried under a stream of N_2_ and resuspended in HPLC grade water. Samples were immediately placed in an Ultimate 3000 RS autosampler (Dionex, Sunnyvale, CA, United States) and an injection volume of 10 μL was separated through a reverse-phase C18 (Synergi 2.5 μm Hydro- RP, 100 Å, 100 × 2.00 mm) liquid chromatography column (Phenomenex, Torrance, CA, United States) kept at 25°C. Solvent A consisted of 97:3 water:methanol, 10 mM tributylamine, and 15 mM acetic acid. Solvent B was 100% methanol. The solvent gradient from 0 to 5 min was 100% A: 0% B, from 5 to 13 min was 80% A: 20% B, from 13 to 15.5 min was 45% A: 55% B, from 15.5 to19 min was 5% A: 95% B, and from 19 to 25 min was 100% A: 0% B with a flow rate of 200 μL/min. The mass spectrometer was operated in full scan mode following an adopted protocol (Lu et al., 2010). The chromatographic eluent was ionized *via* an electrospray ionization (ESI) source in negative mode and coupled to an Exactive Plus Orbitrap mass spectrometer (Thermo Fisher Scientific, Waltham, MA, United States) through a 0.1-mm internal diameter fused silica capillary tube. The samples were run with a spray voltage of 3 kV, N_2_ sheath gas of 10 (arbitrary units), capillary temperature of 320°C, and automatic gain control (AGC) target set to 3 × 10^6^ ions. Samples were analyzed at a resolution of 140,000 and a scan window of 85–800 *m/z* from 0 to 9 min, and 110–1000 *m/z* from 9 to 25 min. Files generated by Xcalibur (RAW) were converted to the open-source mzML format (Martens et al., 2011) *via* the open-source msconvert software as part of the ProteoWizard package (Chambers et al., 2012). MAVEN (mzroll) software (Melamud et al., 2010; Clasquin et al., 2012), which uses a peak grouping algorithm and retention time alignment, was used for visualization of extracted ion chromatograms. Metabolites, validated from an in-house library of approximately 300 compounds, were first manually identified using samples from the unlabeled nutrient bottles and integrated based on exact mass (± 10 ppm mass tolerance) and retention time (Δ ≤ 1.5 min). Peak intensities for a given sample were normalized by the sum of all metabolites detected for that sample. However, only metabolites that showed stable isotope incorporation were considered in further analysis, ensuring that the metabolites considered were being actively used or produced by the cell at the time of sampling. The metabolomics profiling has been submitted to MetaboLights database^1^ under study MTBLS3715.

### Statistical Analyses

All statistical analyses were carried out in R studio (Rstudio_Team, 2019). Difference between treatments for chlorophyll *a*, toxin concentrations, transcript expression, and metabolite peak intensities was determined using ordinary one-way ANOVAs with a Tukey’s HSD test. A significance level of α = 0.05 was used in all analyses. Shifts in microbial communities for phototrophs and heterotrophs were characterized using canonical correspondence analyses (CCA) that incorporated dissolved nutrient metadata. To better characterize the co-occurring community, *Microcystis* was not included in this analysis. Phylogenetic groups were classified as primary or secondary producers manually. The CCAs were performed using the Vegan package (v. 2.5-7) with the species scores scaled by eigenvalues (Okansen et al., 2020). Pearson correlation coefficients were established the Hmisc package (v. 4.4-2) (Harrell, 2020) with Benjamini–Hochberg corrections for multiple comparisons using the R stats package (v. 3.6.1).

## Results

### Basic Bloom Characterization

**FIGURE 1:**
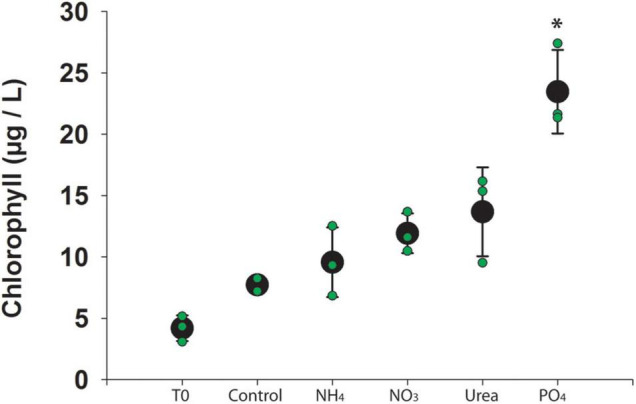
Chlorophyll *a* concentration across experimental manipulations. Individual bottles are denoted in green and the average is denoted in black. Error bars represent standard deviation and star (*) indicates significant difference from control (*p* < 0.05).

Water column physiochemistry at the onset of the experiment included dissolved oxygen = 8.73 mg L^–1^; pH = 8.89; turbidity = 0.63 NTU; temperature = 26.2°C; and chlorophyll *a* = 0.23 RFU. After 48 h of incubation, chlorophyll *a* concentrations were higher in phosphate-amended bottles (*p*-value < 0.001) than in the control bottles (Figure 1 and Supplementary Data 1). Dissolved nutrients revealed a significant drawdown in nitrate in the phosphate-amended bottles (*p*-value < 0.001), as compared to the control bottles (Supplementary Data 1).

### *Microcystis* Nutrient Transport Gene Expression

As expected, *Microcystis* was the most represented genus in the sequencing dataset, with normalized DNA-dependent RNA polymerase (*rpoB*) expression ranging from 5.2 to 27 per 10^7^ base pairs (Supplementary Figure 2). Overall, ammonium (*amt*) and urea transporters (*urt* family) were significantly upregulated relative to the control in the phosphate-amended bottles, while phosphate transporters (*pst* family) were significantly downregulated (*p*-value < 0.01) (Supplementary Data 1). Relative to control, nitrate/nitrite transporters (*nrt* family) were downregulated in the ammonia- and urea-amended bottles (*p*-value < 0.001). Within control bottles, there were significant differences in nitrate and urea transporter expression relative to the T0 samples. Both transporters were significantly upregulated in the control incubations relative to T0 (*p*-values < 0.001) (Supplementary Data 1).

### Co-occurring Host Community Gene Expression

**FIGURE 2:**
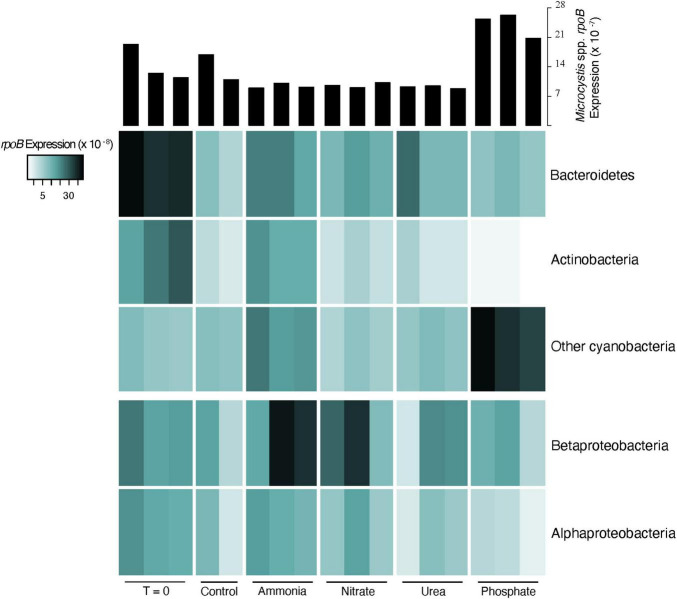
Heatmap of normalized expression of the DNA-dependent RNA polymerase (*rpoB*) of dominant phyla (top three) and classes (bottom two) in co-occurring heterotrophic microbial community. Expression was normalized to the library size for each sample. Histograms at the top describe the transcriptional response of *Microcystis* spp. *rpoB* for comparison.

The sum of *rpoB* expression for all other co-occurring, non-viral organisms in the community ranged from 5.6 to 13 per 10^7^ base pairs (Supplementary Figure 2). In most treatments, *Microcystis* alone showed higher transcript expression levels than the rest of the community combined, particularly in the phosphate treatments. Ammonia was the only treatment in which the combined community transcript expression of *rpoB* was higher than *Microcystis*. Changes in the co-occurring community appeared to be strongly driven by shifts in transcription within the heterotrophic community. This was particularly apparent in the transcriptional shifts in the heterotrophic community observed between T0 and controls (Supplementary Figure 3). We note that this shift was not observed in the ammonia bottles, with the heterotrophic community demonstrating a transcriptional composition more similar to the T0. Characterization of the taxonomy within these shifts confirmed that they primarily occurred in the heterotrophic community, with lesser change in the cyanobacterial species. However, there was notable changes in cyanobacteria between T0 and P-amendment bottles (see below). The majority of active expression (75–85%) in the co-occurring community, based on the sum of all samples, originated from Actinobacteria, Bacteroidetes, Proteobacteria, and other Cyanobacteria species (Figure 2). Excluding *Microcystis*, Bacteroidetes showed the highest levels of active transcription in the T0 bottles, while Betaproteobacteria was the most transcriptionally active in the ammonia and nitrate bottles. Within the cyanobacterial group (excluding *Microcystis*), *Pseudanabaena* was the most transcriptionally active in all but the ammonia bottles, where *Synechococcaceae* were the most represented (Supplementary Data 1). Variations in dominant co-occurring taxa occur not only between nutrient treatments, but among bottles within the same treatment. For example, there is notable variability in Betaproteobacteria between replicates. Even when *Microcystis* remained fairly constant between replicates of a treatment (*e.g.*, ammonia, nitrate, and urea additions), the co-occurring microbial community did not (Figure 2).

### Viral Community Characterization

**FIGURE 3:**
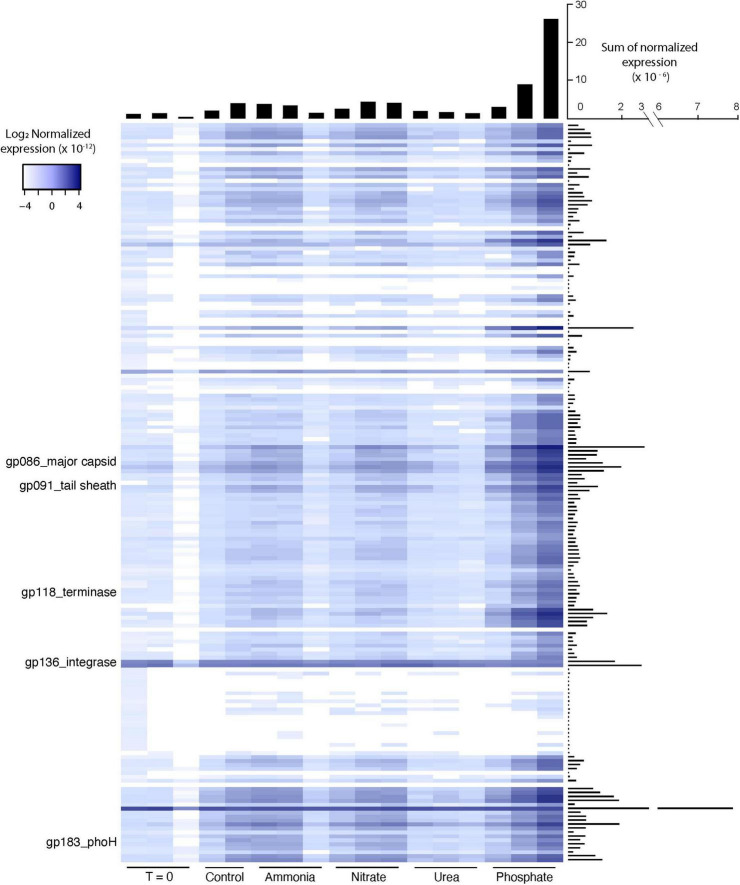
Heatmap of normalized expression for *Microcystis* phage Ma-LMM01 genes in individual experimental bottles. Histograms describe the sum of normalized expression for each bottle (horizontal histogram) and each gene (vertical histogram). Expression was normalized to the library size of each sample and the length of each gene.

An evaluation of the viral community using a BLASTx approach revealed that *Microcystis* phage were the most transcriptionally active viruses and that viruses infecting other community members were either largely absent or were not actively transcribed. Normalized expression computed from either *Microcystis* phage contigs (data not shown) or from the entire *Microcystis* phage Ma-LMM01 genome indicated low levels of phage activity (2.2–57 per 10^8^ base pair) in most bottles, excluding the phosphate amendments. However, across phosphate treatments, phage expression was highly variable, ranging from 4.7 to 48 per 10^7^ base pair (Figure 3). The third phosphate replicate had a 10-fold increase in phage transcript expression compared to the first phosphate replicate. These increases were not associated with changes in host expression, as *Microcystis rpoB* transcription remained unchanged between +P and other treatments.

**FIGURE 4:**
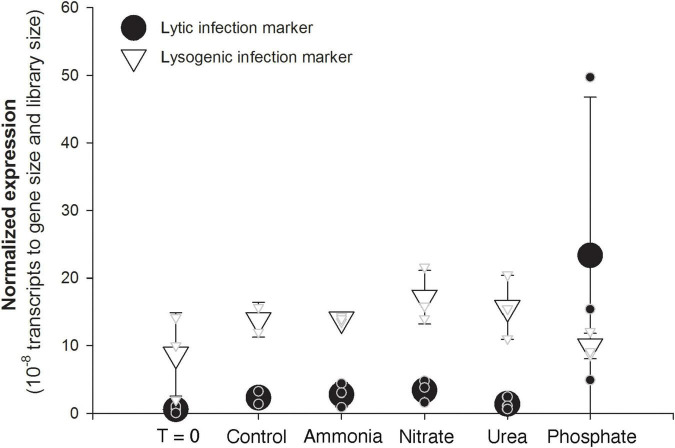
Average (large symbols) and individual bottle (small symbols) normalized expression of a *Microcystis* phage marker of lytic activity (gp91 tail sheath, black circles) and a marker of lysogenic activity (gp136 recombinase/integrase, open triangles). Expression was normalized to both library size of each sample and the length of each gene.

The pattern of gene expression across the *Microcystis* phage Ma-LMM01 genome held fairly constant, with most genes following a similar dramatic increase in the third phosphate replicate. This includes genes that serve as typical markers for lytic activity, such as the tail sheath gene (gp_91), the major capsid gene (gp_86), and the terminase gene (gp_118) (Stough et al., 2017; Figure 3). A hypothesized auxiliary metabolic gene thought to be induced by phosphate starvation, *phoH*, also showed the dramatic increase in transcription in the third phosphate replicate relative to all others. In contrast, expression of genes associated with lysogeny, such as the integrase/recombinase (gp_136) and transposase (gp_135), displayed consistent and relatively low transcription across all bottles, including all of the phosphate bottles (Figure 4).

Overall, the BLASTx analysis indicated that transcription of non-*Microcystis* virus genes, either prokaryotic- and eukaryotic-infecting viruses, were nearly absent (data not shown). An exception included a *Synechococcus*-like myovirus terminase gene that was more transcriptionally active than *Microcystis* phage in the T0 and ammonia-amended bottles (Supplementary Figure 4).

### Correlation Between Phage Gene Expression and Metabolites

**FIGURE 5:**
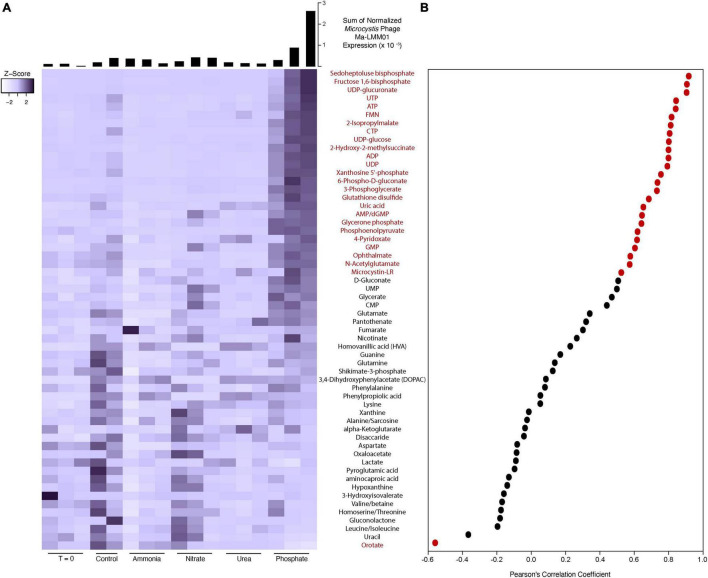
Heatmap of metabolite peak intensities **(A)**. Color scale has been scaled to each metabolite (*z*-score). Histograms describe the sum of normalized expression for *Microcystis* phage Ma-LMM01 in each experimental bottle. Expression was normalized to the library size of each sample and the length of each gene. Scatter plot shows the Pearson’s correlation coefficient between each metabolite detected and *Microcystis* phage Ma-LMM01 tail sheath normalized expression **(B)**. Red dots and metabolite labels indicate a significant correlation (*p* < 0.05), while black dots and metabolite labels indicate correlations deemed to be non-significant statistically (*p* > 0.05).

A total of 58 identified metabolites were detected in the community, all of which were being actively cycled at the time of sampling (Figure 5A). Almost half (24 of 58) of the metabolites were positively correlated to expression of the Ma-LMM01 tail sheath gene (gp_91), an indicator of lytic infection activity [false discovery rate (FDR) corrected *p*-value < 0.05] (Figure 5B). One metabolite (orotate) was inversely correlated to gp_91 expression. Many of the significantly correlated metabolites have been linked to phage expression in studies of other systems, including UDP sugars and metabolites involved in the pentose phosphate pathway (Thompson et al., 2011; Ankrah et al., 2014).

It is unclear what proportion of the metabolite pools can be attributed to *Microcystis* activity. To gain insight into linkages between *Microcystis* and detected *Microcystis* phage, we correlated all *Microcystis* expression to the phage tail sheath (gp_91) expression across all of our samples. Supplementary Figure 5 shows the expression of each KEGG orthology group annotated as *Microcystis*, and many show the same pattern of increased expression in the third phosphate replicate. In fact, 497 of the 1,414 detected (35.1%) *Microcystis* spp. KEGG orthology functional groups were significantly correlated to the *Microcystis* Ma-LMM01 tail sheath gene (FDR *p*-value < 0.05). All of these correlations were positive, with none of the KEGG orthology groups that were negatively correlated (429) meeting statistical significance.

## Discussion

Experiments in microbial ecology often use container-bound populations to explore responses to perturbation. These experiments involve replicate bottles or micro/mesocosms to increase confidence in the observations by reproducing results. When identical treatment bottles, or replicates, do not show identical responses, many scientists consider the discrepancies to be “experimental error” or “unexplained variability.” Variability can come in many forms: for example, in the current study it took up to 2 h to process samples after the 48-h incubation period, which provided for an extended (albeit minimal) incubation time for some samples. Unfortunately, this is a logistical limitation that likely effects all experiments with a well-replicated design. In the current experiments, however, at least some of this variability can potentially be attributed to subtle shifts in the microbiome of the target community, which can occur in either natural systems or in non-axenic lab cultures (Pound et al., 2021a). Moreover, as recently shown in other systems (Kuhlisch et al., 2021; Vincent et al., 2021) we can take advantage of the variability between replicates to learn more about our system.

Cyanobacterial blooms are often characterized by the amount of chlorophyll *a* present. While chlorophyll *a* concentration can provide a useful baseline for bloom biomass, it serves as a non-specific estimate of phototrophs in the community. It does not provide information on the diversity of phototrophic community nor the heterotrophic community. In this study, measurements of chlorophyll *a* response to nutrient additions revealed that the phototrophic community was constrained by available phosphorus. RNA sequence analyses confirmed that transcription in the phototrophic community was dominated by *Microcystis*, particularly in the phosphate-amended bottles. Phosphate constraints on *Microcystis* biomass has been well-documented in many natural bloom systems (Davis et al., 2010). Microcystin concentrations were not different between treatments (*p*-value > 0.1), nor were there differences in congener profile (Supplementary Figure 1). This is in many ways not surprising as it was recently demonstrated that up-regulation of the genes associated with toxin synthesis in *Microcystis* can take 48 h to result in a measurable increase in toxin quota (Martin et al., 2020). There was no significant change in chlorophyll *a* associated with nitrogen amendments, indicating that the phototroph biomass was not constrained by available nitrogen at the time of sampling.

However, as hypothesized nearly two decades ago (Wilhelm et al., 2003), nitrogen-amendments did cause a change in the community transcription. Our canonical correspondence analysis revealed that the microbial community transcriptional shifts occurred primarily within the heterotrophic bacterial community. This was particularly evident in the control and ammonia-amended bottles. The initial heterotrophic bacterial community was dominated by Bacteroidetes transcription, but after 48 h of incubation, the active heterotrophic microbial community in the control bottles had shifted, while the phototrophic community remained largely unchanged. The addition of ammonia appeared to maintain the transcriptional dominance of Bacteroidetes and Betaproteobacteria. This observation suggested that ammonia recycling was decoupled when the *in situ* heterotrophic community was constrained to a bottle. To phrase this another way, it suggests that at the time of sampling, the *in situ* bacterial community structure may have been dependent on ammonia as a main N source. Heterotrophic bacteria generally have faster growth rates than many planktonic phototrophs, which could allow for a more rapid change in that community because of changing environmental pressures (DeBruyn et al., 2004). This shift in community activity in the control bottles is one example of how bottle effects can alter community dynamics before consequences of experimental treatments are ever considered. We note that this response is not unexpected: recently we (Pound et al., 2021a) demonstrated that a single batch culture of non-axenic *Microcystis aeruginosa* undergoes dramatic shifts in the transcriptional activity of co-occurring heterotrophic bacteria after only three batch culture transfers in growth media containing different nitrogen sources.

The hypothesis that the *in situ* community was utilizing ammonia regeneration as a nitrogen source, even in high concentrations of nitrate (initial nitrate concentration was 30 μmol/L), finds support within the *Microcystis* functional gene expression data. The decrease in expression of nitrate/nitrite transporters in the ammonia- and urea-amended bottles suggested these communities were not relying on nitrate or nitrite as a primary nitrogen source. This is further supported by the increase in urea and ammonia transporters observed in the controls, indicating that *Microcystis* was preferentially using those nitrogen sources when enclosed in a bottle. When phosphate was added to some bottles, we saw a similar increase in urea and ammonia transporters, indicating a phosphate-constrained community that showed preferential uptake of urea and ammonia as a nitrogen source. This conclusion is supported by several recent studies. The utilization of regenerated ammonium has been shown to support non-N-fixing cyanobacterial blooms, such as *Microcystis*, in Lake Erie (Hampel et al., 2019), and a stable isotope study showed that *Microcystis* preferentially assimilates low concentrations of ammonium in the presence of high nitrate concentrations (Chaffin and Bridgeman, 2014). A preference for urea has been observed in many other *Microcystis* bloom events: urea is a common agricultural-based anthropogenic input, serving as both a nitrogen and carbon source to bloom organisms (Davis et al., 2010; Krausfeldt et al., 2019).

Characterization of the microbial community, based on *rpoB* transcriptional levels, revealed that bottle effects not only caused shifts between treatments, but can also cause shifts within treatments, resulting in variability between replicate bottles. Once a community is isolated in a container, it undergoes succession independent of other communities/containers regardless of treatment. Heterogeneity in natural microbial systems provides initial variation in bottled communities, and through independent succession, communities diverge further when contained. This phenomenon is apparent in several of the observations made from our experiments. On the simplest level, we recorded variable chlorophyll *a* concentrations between bottles of the same treatment. While such variation is common, the cause is rarely considered nor explained. It could be the result of a physical or chemical change, such as a shift in light levels, or a biological change in the non-target (*i.e.*, in this case the heterotrophic microbial) community. It could either mean a shift in the type of phototroph present, or a shift in the photosynthetic activity of a single taxa, or some combination thereof. While there are a variety of potential sources of variability, shifts in the microbial community, either in function or taxonomy, could play a crucial role in observed variation. We also see bottle effects influencing intra-treatment variability in community composition. We documented variations in the heterotrophic community between bottles of the same treatment, where expression levels of the same group of organisms varied dramatically between bottles.

The most dramatic example of within-treatment variability was observed in transcriptional activity of *Microcystis*-infecting phage in the phosphate-amended bottles. The three replicate bottles showed different levels of phage gene expression, with one replicate displaying 10-fold higher transcriptional activity of *Microcystis*-infecting phage relative to another. These viral expression levels in the third phosphate replicate suggested a significant ongoing lytic infection during incubation. Consistent with this idea, *Microcystis* abundance (as measured by *rpoB* transcription) was ∼25% lower in this sample compared to the other P-amended replicates. In contrast, expression of the genetic marker for lysogeny was consistent and relatively low across all bottles, including the bottle with high transcriptional levels indicating lytic activity. This suggests that the possible lytic event was not the result of lysogenic induction (or at least that any lysogenic induction was incomplete). Such variable virus expression has recently been observed in other experimental systems, including an *Emiliania huxleyi* bloom induced in mesocosms, where researchers noted highly variable virus expression between replicate samples (Kuhlisch et al., 2021). In a likewise manner, their observations were tied to variability in host metrics, including chlorophyll *a* and metabolites.

The occurrence of a large-scale virus-mediated lytic event, even when a host population does not fully lyse, can have massive effects on host dynamics. We have shown that ∼35% of KO functional groups in *Microcystis* were significantly correlated to the phage transcript expression, indicating that the cells were responding to the viral activity. Many of these KO functional groups seemed to be associated with nucleotide recycling and urea utilization. Given that nucleotide recycling is a major component of virus replication, this seems like a rational relationship. We have also shown that ∼45% of the detected cellular metabolites from the entire community were significantly correlated with virus marker expression. The observed high levels of UDP sugars have been previously documented during viral infection of a marine heterotroph (Ankrah et al., 2014). Those authors suggested that these sugars may reflect changes in cell wall integrity as result of virus activity. Other metabolites, such as sedoheptulose bisphosphate and fructose-1,6 bisphosphate, are involved in the pentose phosphate pathways which has been hypothesized to provide extra energy and nucleotide precursors during viral infections of other cyanobacterial species (Thompson et al., 2011). Although we cannot determine the abundance of metabolites produced solely by *Microcystis*, it is possible that the community, including *Microcystis*, is responding to the viral-activity-generated “virocell” state (cf. Forterre, 2011) within this cyanobacterium: as *Microcystis* biochemistry changes due to infection the co-occurring community undergoes some parallel change. We note that not all viruses detected within these experiments demonstrated this variability between replicates. For example, myoviruses infecting *Synechococcus* provided very consistent, reproducible data across replicates, with elevated transcriptional activity observed in the ammonia-amended bottles that also had a higher *Synechococcus* host transcriptional activity (Supplementary Figure 4).

Without the analysis of viral transcription and metabolite pools, the presence of and the variation within the *Microcystis* viral infection could have been overlooked, with the resulting variability going unexplained. Indeed, any resulting phage influence on *Microcystis* transcription might be misconstrued, leading to invalid conclusions on the dynamics of the effect of phosphate. While not as dramatic in the current study, the same conclusions could be erroneously arrived at due to shifts in the function of the heterotrophic community. Functional relationships between *Microcystis* and its associated heterotrophic counterparts are still unclear, but many studies acknowledge a possible mutualistic relationship that can alter *Microcystis* growth dynamics (Kim et al., 2019; Cook et al., 2020). The “minor” community shifts that occur due to bottle effects have the potential to cause functional changes in *Microcystis*, introducing variability that might otherwise be ignored or left unexplained. Indeed, the apparent reliance on ammonium regeneration within the heterotrophic community could be a source of such variability within a 48-h timescale. To this end, researchers need to carefully consider the dynamics of any incubation experiments they undertake, as it is highly likely that this divergence due to “unintended biology” increases with length of time over any incubation period.

One surprising observation in this data set is the lack of transcripts assigned to non-*Microcystis*-infecting phage. Viruses are common components of metagenomic (*i.e.*, DNA) sequencing efforts, with phage commonly having significant representation. This is expected given estimates of 10^5^–10^7^ phage per milliliter of water (Wilhelm and Matteson, 2008; Wigington et al., 2017). Yet in transcriptional analyses of several systems (e.g., Stough et al., 2018; Pound et al., 2020), we have observed that transcripts that can be assigned to phage appear more limited than expected. Given that our marker gene approach can find phage in DNA (genomic) samples, it is surprising that the same marker gene approach does not detect a higher expression of phage transcripts. In the current study, we again see phage contributing a surprisingly limited part of the transcriptome: while transcripts from phage infecting *Microcystis* are abundant, all other phage transcripts account for only a minimal component of library reads. While many scenarios might explain these observations, the most parsimonious would be that phage infection of the co-occurring community remains rather limited. While the idea of phage infecting the most abundant host is not new (Murray and Jackson, 1993; Thingstad and Lignell, 1997; Wilhelm et al., 1998), the extremely low abundance of phage transcripts would seem to fly in the face of past ideas on the importance of viruses in systems ecology (Wilhelm and Suttle, 1999) and perhaps requires further examination.

The complexity of biological systems cannot be overstated. Each organism (including viruses, heterotrophic bacteria, cyanobacteria, eukaryotic microalgae, and fungi) can play a pivotal role in cyanobacterial bloom dynamics. Going forward, this work demonstrates a need to broadly consider the influence of each entity in the ecosystem under study. Moreover, casting light on variability within basic experimental methods will not only provide a better understanding of the community of interest, but of biology (and all its possibilities) in general.

## Data Availability Statement

The datasets presented in this study can be found in online repositories. Repository names and accession number(s) are provided in the article and the Supplementary Material.

## Author Contributions

HP and SW designed the experiments. HP, BZ, RM, and JC completed the sample collection and the experimental incubations. HP and RM processed the samples. CC, SH, HC, and SC performed the mass spectrometry for the metabolomes. HP analyzed the transcriptomes and metabolomes. All authors participated in development of the manuscript and manuscript revision.

## Conflict of Interest

The authors declare that the research was conducted in the absence of any commercial or financial relationships that could be construed as a potential conflict of interest.

## Publisher’s Note

All claims expressed in this article are solely those of the authors and do not necessarily represent those of their affiliated organizations, or those of the publisher, the editors and the reviewers. Any product that may be evaluated in this article, or claim that may be made by its manufacturer, is not guaranteed or endorsed by the publisher.

## Footnotes

1
https://www.ebi.ac.uk/metabolights

## References

[B1] Ankrah N. Y. D. May A. L. Middleton J. L. Jones D. R. Hadden M. K. Gooding J. R. (2014). Phage infection of an environmentally relevant marine bacterium alters host metabolism and lysate composition. *ISME J.* 8 1089–1100. 10.1038/ismej.2013.216 24304672 PMC3996693

[B2] Belisle B. S. Steffen M. M. Pound H. L. Watson S. B. DeBruyn J. M. Bourbonniere R. A. (2016). Urea in lake erie: organic nutrient sources as potentially important drivers of phytoplankton biomass. *J. Great Lakes Res.* 42 599–607. 10.1016/j.jglr.2016.03.002

[B3] Besemer J. Borodovsky M. (1999). Heuristic approach to deriving models for gene finding. *Nucleic Acids Res.* 27 3911–3920. 10.1093/nar/27.19.3911 10481031 PMC148655

[B4] Boyer G. L. (2020). *LCMS-SOP Determination Of Microcystins In Water Samples By High Performance Liquid Chromatography (HPLC) With Single Quadrupole Mass Spectrometry (MS) [Online]. Protocols.io.* Available online at: 10.17504/protocols.io.bck2iuye (accessed January 18, 2022).

[B5] Breitbart M. (2012). Marine viruses: truth or dare. *Ann. Rev. Mar. Sci.* 4 425–448. 10.1146/annurev-marine-120709-142805 22457982

[B6] Camacho C. Coulouris G. Avagyan V. Ma N. Papadopoulos J. Bealer K. (2009). BLAST+: architecture and applications. *BMC Bioinformatics* 10:421. 10.1186/1471-2105-10-421 20003500 PMC2803857

[B7] Carpenter S. R. Chisholm S. W. Krebs C. J. Schindler D. W. Wright R. F. (1995). Ecosystem experiments. *Science* 269 324–327. 17841247 10.1126/science.269.5222.324

[B8] Chaffin J. D. Bridgeman T. B. (2014). Organic and inorganic nitrogen utilization by nitrogen-stressed cyanobacteria during bloom conditions. *J. Appl. Phycol.* 26 299–309. 10.1007/s10811-013-0118-0

[B9] Chaffin J. D. Mishra S. Kane D. D. Bade D. L. Stanislawczyk K. Slodysko K. N. (2019). Cyanobacterial blooms in the central basin of Lake Erie: potentials for cyanotoxins and environmental drivers. *J. Great Lakes Res.* 45 277–289.

[B10] Chambers M. C. Maclean B. Burke R. Amodei D. Ruderman D. L. Neumann S. (2012). A cross-platform toolkit for mass spectrometry and proteomics. *Nature Biotechnol.* 30 918–920. 10.1038/nbt.2377 23051804 PMC3471674

[B11] Clasquin M. F. Melamud E. Rabinowitz J. D. (2012). LC-MS data processing with MAVEN: a metabolomic analysis and visualization engine. *Curr. Protocols Bioinformatics* 37 14.11.11–14.11.23. 10.1002/0471250953.bi1411s37 22389014 PMC4055029

[B12] Cook K. V. Li C. Cai H. Krumholz L. R. Hambright K. D. Paerl H. W. (2020). The global *Microcystis* interactome. *Limnol. Oceanogr.* 65 S194–S207. 10.1002/lno.11361 32051648 PMC7003799

[B13] Davidson E. A. Howarth R. W. (2007). Environmental science: nutrients in synergy. *Nature* 449, 1000–1001. 10.1038/4491000a 17960233

[B14] Davis T. W. Harke M. J. Marcoval M. A. Goleski J. Orano-Dawson C. Berry D. L. (2010). Effects of nitrogenous compounds and phosphorus on the growth of toxic and non-toxic strains of *Microcystis* during cyanobacterial blooms. *Aquat. Microb. Ecol.* 61 149–162.

[B15] DeBruyn J. M. Leigh-Bell J. A. McKay R. M. L. Bourbonniere R. A. Wilhelm S. W. (2004). Microbial distributions and the impact of phosphorus on bacterial activity in Lake Erie. *J. Great Lakes Res.* 30 166–183. 10.1016/s0380-1330(04)70338-x

[B16] Forterre P. (2011). Manipulation of cellular synthases and the nature of viruses: the virocell concept. *Compets Rendus Chim.* 14 392–399.

[B17] Fuhrman J. A. (1999). Marine viruses and their biogeochemical and ecological effects. *Nature* 399 541–548. 10376593 10.1038/21119

[B18] Guindon S. Dufayard J.-F. Lefort V. Anisimova M. Hordijk W. Gascuel O. (2010). New algorithms and methods to estimate maximum-likelihood phylogenies: assessing the performance of PhyML 3.0. *Syst. Biol.* 59 307–321. 10.1093/sysbio/syq010 20525638

[B19] Hampel J. J. Mccarthy M. J. Neudeck M. Bullerjahn G. S. McKay R. M. L. Newell S. E. (2019). Ammonium recycling supports toxic *Planktothrix* blooms in Sandusky Bay, Lake Erie: evidence from stable isotope and metatranscriptome data. *Harmful Algae* 81 42–52. 10.1016/j.hal.2018.11.011 30638497

[B20] Harrell F. E. Jr. (2020). *Hmisc: Harrell Miscellaneous. R Package Version 4.4-2.* Available online at: https://CRAN.R-project.org/package=Hmisc (accessed November 5, 2021).

[B21] Hutchins D. A. Franck V. M. Brzezinski M. A. Bruland K. W. (1999). Inducing phytoplankton iron limitation in iron-replete coastal waters with a strong chelating agent. *Limnol. Oceanogr.* 44 1009–1018.

[B22] Jankowiak J. G. Gobler C. J. (2020). The composition and function of microbiomes within *Microcystis* colonies are significantly different than native bacterial assemblages in two North American Lakes. *Front. Microbiol.* 11:1016. 10.3389/fmicb.2020.01016 32547511 PMC7270213

[B23] Kanehisa M. Sato Y. Morishima K. (2016). BlastKOALA and GhostKOALA: KEGG tools for functional characterization of genome and metagenome sequences. *J. Mol. Biol.* 428 726–731. 10.1016/j.jmb.2015.11.006 26585406

[B24] Kim M. Shin B. Lee J. Park H. Y. Park W. (2019). Culture-independent and culture-dependent analyses of the bacterial community in the phycosphere of cyanobloom-forming *Microcystis aeruginosa*. *Sci. Rep.* 9 1–13. 10.1038/s41598-019-56882-1 31892695 PMC6938486

[B25] Kopylova E. Noé L. Touzet H. (2012). SortMeRNA: fast and accurate filtering of ribosomal RNAs in metatranscriptomic data. *Bioinformatics* 28 3211–3217. 10.1093/bioinformatics/bts611 23071270

[B26] Krausfeldt L. E. Farmer A. T. Gonzalez H. C. Zepernick B. N. Campagna S. R. Wilhelm S. W. (2019). Urea is both a carbon and nitrogen source for *Microcystis aeruginosa*: tracking 13C incorporation at bloom pH conditions. *Front. Microbiol.* 10:1064. 10.3389/fmicb.2019.01064 31164875 PMC6536089

[B27] Kuhlisch C. Schleyer G. Shahaf N. Vincent F. Schatz D. Vardi A. (2021). Viral infection of algal blooms leaves a unique metabolic footprint on the dissolved organic matter in the ocean. *Sci. Adv.* 7:eabf4680. 10.1126/sciadv.abf4680 34144983 PMC8213229

[B28] Li D. Liu C.-M. Luo R. Sadakane K. Lam T.-W. (2015). MEGAHIT: an ultra-fast single-node solution for large and complex metagenomics assembly via succinct de Bruijn graph. *Bioinformatics* 31 1674–1676. 10.1093/bioinformatics/btv033 25609793

[B29] Lindell D. Jaffe J. D. Johnson Z. I. Church G. M. Chisholm S. W. (2005). Photosynthesis genes in marine viruses yield proteins during host infection. *Nature* 438 86–89. 10.1038/nature04111 16222247

[B30] Love M. I. Huber W. Anders S. (2014). Moderated estimation of fold change and dispersion for RNA-seq data with DESeq2. *Genome Biol.* 15 1–21. 10.1186/s13059-014-0550-8 25516281 PMC4302049

[B31] Lu W. Clasquin M. F. Melamud E. Amador-Noguez D. Caudy A. A. Rabinowitz J. D. (2010). Metabolomic analysis *via* reversed-phase ion-pairing liquid chromatography coupled to a stand alone orbitrap mass spectrometer. *Anal. Biochem.* 82 3212–3221. 10.1021/ac902837x 20349993 PMC2863137

[B32] Martens L. Chambers M. Sturm M. Kessner D. Levander F. Shofstahl J. (2011). mzML—a community standard for mass spectrometry data. *Mol. Cell. Proteomics* 10:R110.000133. 10.1074/mcp.R110.000133 20716697 PMC3013463

[B33] Martin J. H. Fitzwater S. E. (1988). Iron deficiency limits phytoplankton growth in the north-east Pacific subarctic. *Nat.* 331 341–343. 10.1038/331341a0

[B34] Martin R. M. Moniruzzaman M. Stark G. F. Gann E. R. Derminio D. S. Wei B. (2020). Episodic decrease in temperature increases mcy gene transcription and cellular microcystin in continuous cultures of *Microcystis aeruginosa* PCC 7806. *Front. Microbiol.* 11:601864. 10.3389/fmicb.2020.601864 33343544 PMC7744600

[B35] Matsen F. A. Kodner R. B. Armbrust E. V. (2010). pplacer: linear time maximum-likelihood and Bayesian phylogenetic placement of sequences onto a fixed reference tree. *BMC Bioinformatics* 11:538. 10.1186/1471-2105-11-538 21034504 PMC3098090

[B36] Melamud E. Vastag L. Rabinowitz J. D. (2010). Metabolomic analysis and visualization engine for LC- MS data. *Anal. Chem.* 82 9818–9826. 10.1021/ac1021166 21049934 PMC5748896

[B37] Murray A. G. Jackson G. A. (1993). Viral dynamics II: a model of the interaction of ultraviolet light and mixing processes on virus survival in seawater. *Mar. Ecol. Prog. Ser.* 102 105–114. 10.3354/meps095105

[B38] Okansen J. Blanchet F. G. Friendly M. Kindt R. Legendre P. Mcglinn D. (2020). *vegan: Community Ecology Package. R Package Version 2.5-7.* Available online at: https://CRAN.R-project.org/package=vegan (accessed November 5, 2021).

[B39] Popper K. (1959). *The Logic of Scientific Discovery.* Abingdon-on-Thames: Routledge.

[B40] Pound H. L. Gann E. R. Tang X. Krausfeldt L. E. Huff M. Staton M. E. (2020). The “neglected viruses” of Taihu: abundant transcripts for viruses infecting eukaryotes and their potential role in phytoplankton succession. *Front. Microbiol.* 11:388. 10.3389/fmicb.2020.00338 32210938 PMC7067694

[B41] Pound H. L. Martin R. M. Sheik C. S. Steffen M. M. Newell S. E. Dick G. J. (2021a). Environmental studies of cyanobacterial harmful algal blooms should include interactions with the dynamic microbiome. *Environ. Sci. Technol.* 55 12276–12279. 10.1021/acs.est.1c04207 34529413 PMC9017748

[B42] Pound H. L. Wilhelm S. W. (2019). *Metatranscriptomic Screening for Genes of Interest [online]. Protocols.io.* Available online at: 10.17504/protocols.io.7vyhn7w (accessed January 21, 2022).

[B43] Pound H. L. Wilhelm S. W. (2020a). *RNA Extraction from Sterivex Using Phenol:Chloroform [Online]. Protocols.io.* Available online at: 10.17504/protocols.io.bhu6j6ze (accessed January 20, 2022).

[B44] Pound H. L. Wilhelm S. W. (2020b). Tracing the active genetic diversity of *Microcystis* and *Microcystis* phage through a temporal survey of Taihu. *PLoS One* 15:e0244482. 10.1371/journal.pone.0244482 33370358 PMC7769430

[B45] Pound H. L. Wilhelm S. W. (2021b). *Sequence Processing and Assembly Workflow Using CLC Workbench, SortMeRNA, and MegaHit [Online]. Protocols.io.* Available online at: 10.17504/protocols.io.buvdnw26 (accessed January 21, 2022).

[B46] Pound H. L. Gann E. R. Wilhelm S. W. (2021b). *Functional and Taxonomic Characterization of Sequence Data Using GhostKOALA [Online]. Protocols.io.* Available online at: 10.17504/protocols.io.buvbnw2n (accessed January 20, 2022).

[B47] Roux S. Hallam S. J. Woyke T. Sullivan M. B. (2015). Viral dark matter and virus–host interactions resolved from publicly available microbial genomes. *eLife* 4:e08490. 10.7554/eLife.08490 26200428 PMC4533152

[B48] Rstudio_Team (2019). *RStudio: Integrated, Development of R.* Boston, MA: Rstudio_Team.

[B49] Schelske C. (1984). “In situ and natural phytoplankton assemblage bioassays,” in *Algae As Ecological Indicators*, ed. Shubert L. E. (Cambridge: Academic press), 15–47.

[B50] Schindler D. W. (1998). Whole-ecosystem experiments: replication versus realism: the need for ecosystem-scale experiments. *Ecosystems* 1 323–334. 10.1007/s100219900026

[B51] Spivak A. C. Vanni M. J. Mette E. M. (2011). Moving on up: can results from simple aquatic mesocosm experiments be applied across broad spatial scales? *Freshw. Biol.* 56 279–291. 10.1111/j.1365-2427.2010.02495.x

[B52] Steffen M. M. Davis T. W. McKay R. M. Bullerjahn G. S. Krausfeldt L. E. Stough J. M. A. (2017). Ecophysiological examination of the Lake Erie *Microcystis* bloom in 2014: linkages between biology and the water supply shutdown of Toledo, OH. *Environ. Sci. Technol.* 51 6745–6755. 10.1021/acs.est.7b00856 28535339

[B53] Sterner R. W. (2008). On the phosphorus limitation paradigm for lakes. *Int. Rev. Hydrobiol.* 93 433–445. 10.1002/iroh.200811068

[B54] Stewart F. J. Dalsgaard T. Young C. R. Thamdrup B. Revsbech N. P. Ulloa O. (2012). Experimental incubations elicit profound changes in community transcription in OMZ bacterioplankton. *PLoS One* 7:e37118. 10.1371/journal.pone.0037118 22615914 PMC3353902

[B55] Stough J. M. A. Kolton M. Kostka J. E. Weston D. J. Pelletier D. A. Wilhelm S. W. (2018). Diversity of active viral infections within the *Sphagnum* microbiome. *Appl. Environ. Microbiol.* 84:e01124–18. 10.1128/AEM.01124-18 30217851 PMC6238052

[B56] Stough J. M. Tang X. Krausfeldt L. E. Steffen M. M. Gao G. Boyer G. L. (2017). Molecular prediction of lytic *vs* lysogenic states for *Microcystis* phage: metatranscriptomic evidence of lysogeny during large bloom events. *PLoS One* 12:e0184146. 10.1371/journal.pone.0184146 28873456 PMC5584929

[B57] Sullivan M. B. Weitz J. S. Wilhelm S. W. (2017). Viral ecology comes of age. *Environ. Microbiol. Rep.* 9 33–35. 10.1111/1758-2229.12504 27888577

[B58] Tang X. Krausfeldt L. E. Shao K. Lecleir G. R. Stough J. M. Gao G. (2018). Seasonal gene expression and the ecophysiological implications of toxic *Microcystis aeruginosa* blooms in Lake Taihu. *Environ. Sci. Technol.* 52 11049–11059. 10.1021/acs.est.8b01066 30168717

[B59] Thingstad T. F. Lignell R. (1997). Theoretical models for the control of bacterial growth rate, abundance, diversity and carbon demand. *Aquat. Microb. Ecol.* 13 19–27. 10.3354/ame013019

[B60] Thompson L. R. Zeng Q. Kelly L. Huang K. H. Singer A. U. Stubbe J. (2011). Phage auxiliary metabolic genes and the redirection of cyanobacterial host carbon metabolism. *Proc. Natl. Acad. Sci. U.S.A.* 108 E757–E764. 10.1073/pnas.1102164108 21844365 PMC3182688

[B61] Urrutia-Cordero P. Ekvall M. K. Hansson L. A. (2015). Responses of cyanobacteria to herbivorous zooplankton across predator regimes: who mows the bloom? *Freshw. Biol.* 60 960–972. 10.1111/fwb.12555

[B62] Vanni M. J. Temte J. (1990). Seasonal patterns of grazing and nutrient limitation of phytoplankton in a eutrophic lake. *Limnol. Oceanogr.* 35 697–709. 10.4319/lo.1990.35.3.0697

[B63] Venter J. C. Remington K. Heidelberg J. F. Halpern A. L. Rusch D. Eisen J. A. (2004). Environmental genome shotgun sequencing of the Sargasso Sea. *Science* 304 66–74. 10.1126/science.1093857 15001713

[B64] Vincent F. Sheyn U. Porat Z. Schatz D. Vardi A. (2021). Visualizing active viral infection reveals diverse cell fates in synchronized algal bloom demise. *Proc. Natl. Acad. Sci. U.S.A.* 118:e2021586118. 10.1073/pnas.2021586118 33707211 PMC7980383

[B65] Welschmeyer N. A. (1994). Fluorometric analysis of chlorophyll a in the presence of chlorophyll b and pheopigments. *Limnol. Oceanogr.* 39 1985–1992. 10.4319/lo.1994.39.8.1985

[B66] Wigington C. H. Sonderegger D. Brussaard C. P. D. Buchan A. Finke J. F. Fuhrman J. A. (2017). Re-examination of the relationship between marine virus and microbial cell abundances (vol 1, pg 15024, 2016). *Nat. Microbiol.* 2:1571. 10.1038/nmicrobiol.2015.24 27572161

[B67] Wilhelm S. W. Matteson A. R. (2008). Freshwater and marine virioplankton: a brief overview of commonalities and differences. *Freshw. Biol.* 53 1076–1089. 10.1111/j.1365-2427.2008.01980.x

[B68] Wilhelm S. W. Suttle C. A. (1999). Viruses and nutrient cycles in the sea. *BioScience* 49 781–788. 10.2307/1313569

[B69] Wilhelm S. W. Brigden S. M. Suttle C. A. (2002). A dilution technique for the direct measurement of viral production: a comparison in stratified and tidally mixed coastal waters. *Microb. Ecol.* 43 168–173. 10.1007/s00248-001-1021-9 11984638

[B70] Wilhelm S. W. DeBruyn J. M. Gillor O. Twiss M. R. Livingston K. Bourbonniere R. A. (2003). Effect of phosphorus amendments on present day plankton communitites in pelagic Lake Erie. *Aquat. Microb. Ecol.* 32 275–285. 10.3354/ame032275

[B71] Wilhelm S. W. Weinbauer M. G. Suttle C. A. Jeffrey W. H. (1998). The role of sunlight in the removal and repair of viruses in the sea. *Limnol. Oceanogr.* 43 586–592. 10.4319/lo.1998.43.4.0586

[B72] Xu H. Paerl H. W. Qin B. Zhu G. H. Gao G. (2010). Nitrogen and phosphorus inputs control phytoplankton growth in eutrophic Lake Taihu, China. *Limnol. Oceanogr.* 55 420–432. 10.4319/lo.2010.55.1.0420

[B73] Yoshida M. Yoshida T. Kashima A. Takashima Y. Hosoda N. Nagasaki K. (2008). Ecological dynamics of the toxic bloom-forming cyanobacterium *Microcystis aeruginosa* and its cyanophages in freshwater. *Appl. Environ. Microbiol.* 74 3269–3273. 10.1128/AEM.02240-07 18344338 PMC2394914

[B74] Yoshida M. Yoshida T. Takashima Y. Kashima A. Hiroishi S. (2010). Real-time PCR detection of host-mediated cyanophage gene transcripts during infection of a natural *Microcystis aeruginosa* population. *Microbes Environ.* 25 211–215. 10.1264/jsme2.me10117 21576874

[B75] Yoshida T. Nagasaki K. Takashima Y. Shirai Y. Tomaru Y. Takao Y. (2008). Ma-LMM01 infecting toxic *Microcystis aeruginosa* illuminates diverse cyanophage genome strategies. *J. Bacteriol.* 190 1762–1772. 10.1128/JB.01534-07 18065537 PMC2258655

[B76] Zhu W. Lomsadze A. Borodovsky M. (2010). *Ab initio* gene identification in metagenomic sequences. *Nucleic Acids Res.* 38:e132. 10.1093/nar/gkq275 20403810 PMC2896542

[B77] Zobell C. E. Anderson D. Q. (1936). Observations on the multiplication of bacteria in different volumes of stored sea water and the influence of oxygen tension and solid surfaces. *Biol. Bull.* 71 324–342. 10.2307/1537438

