# Supplementary material for: Changes in Microbiome Activity and Sporadic Viral Infection Help Explain Observed Variability in Microcosm Studies
Source: Front Microbiol. 2022 Mar 16;13:809989. doi: 10.3389/fmicb.2022.809989 (PMC8966487; doi:10.3389/fmicb.2022.809989)
Supplement: Supplementary file 2 [file Data_Sheet_2.pdf]

Supplemental figures to be published online with

CHANGES IN MICROBIOME ACTIVITY AND SPORADIC VIRAL INFECTION HELP EXPLAIN OBSERVED VARIABILITY IN MICROCOSM STUDIES

Helena L. Pound[1], Robbie M. Martin[1], Brittany N. Zepernick[1], Courtney J. Christopher[2], Sara M. Howard[2], Hector F. Castro[2], Shawn R. Campagna[2], Gregory L. Boyer[3], George S. Bullerjahn[4], Justin D. Chaffin[5] and Steven W. Wilhelm [1*]

[1] Department of Microbiology, The University of Tennessee, Knoxville, TN, 37996, USA

[2] Biological and Small Molecule Mass Spectrometry Core (BSMMSC), The University of Tennessee, Knoxville, TN, 37996, USA

[3] Department of Chemistry, State University of New York, College of Environmental Science and Forestry, Syracuse, NY, USA.

[4] Department of Biological Sciences, Bowling Green State University, Bowling Green, OH 43403, USA

[5] Stone Laboratory and Ohio Sea Grant, The Ohio State University, Put-In-Bay, OH 43456, USA

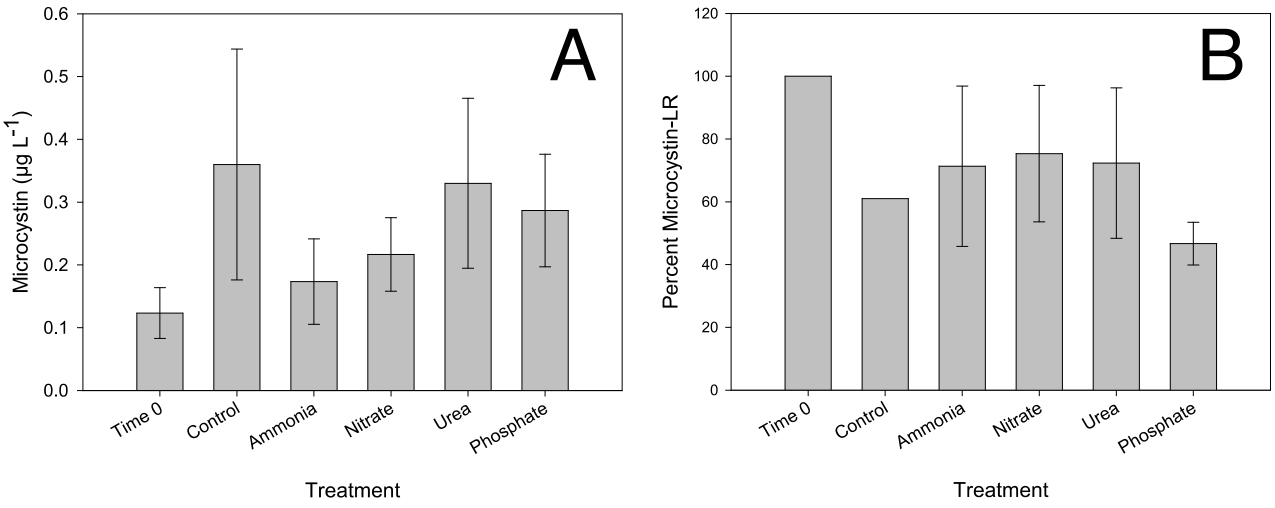

Supplemental Figure 1. Average micrograms of microcystin per liter in each treatment (A). Average percentage of microcystin-LR in each treatment (B). Error bars represent standard deviation.

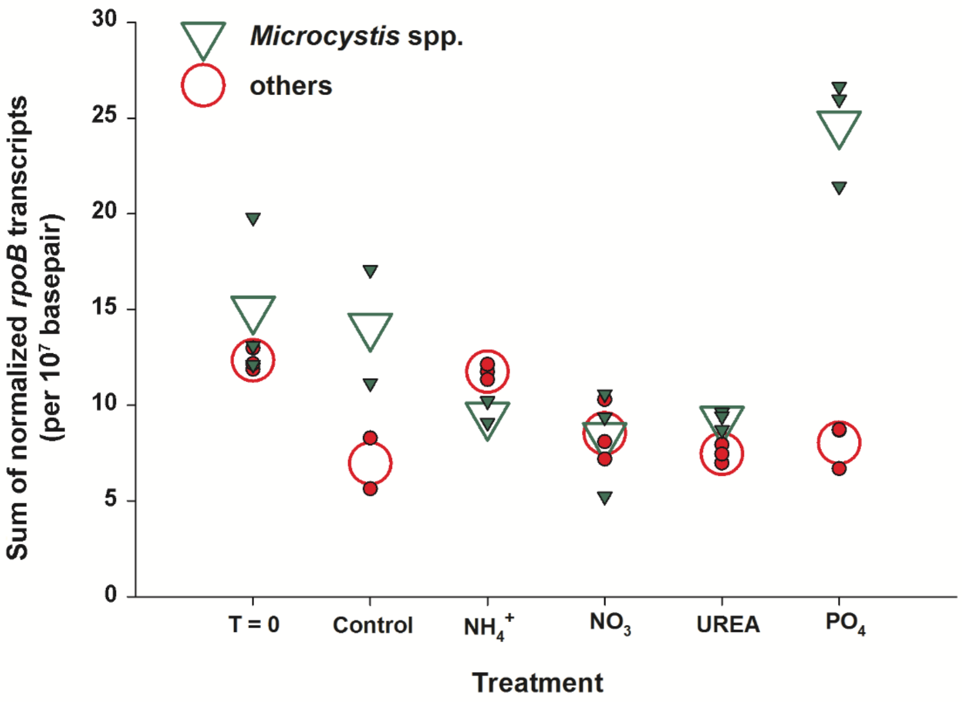

Supplemental Figure 2. Average (large markers) and individual bottle (small markers) expression of normalized expression of the DNA-dependent RNA polymerase (*rpoB*) of *Microcystis* spp. (blue triangles) and the sum of all other co-occurring community members (red circles).

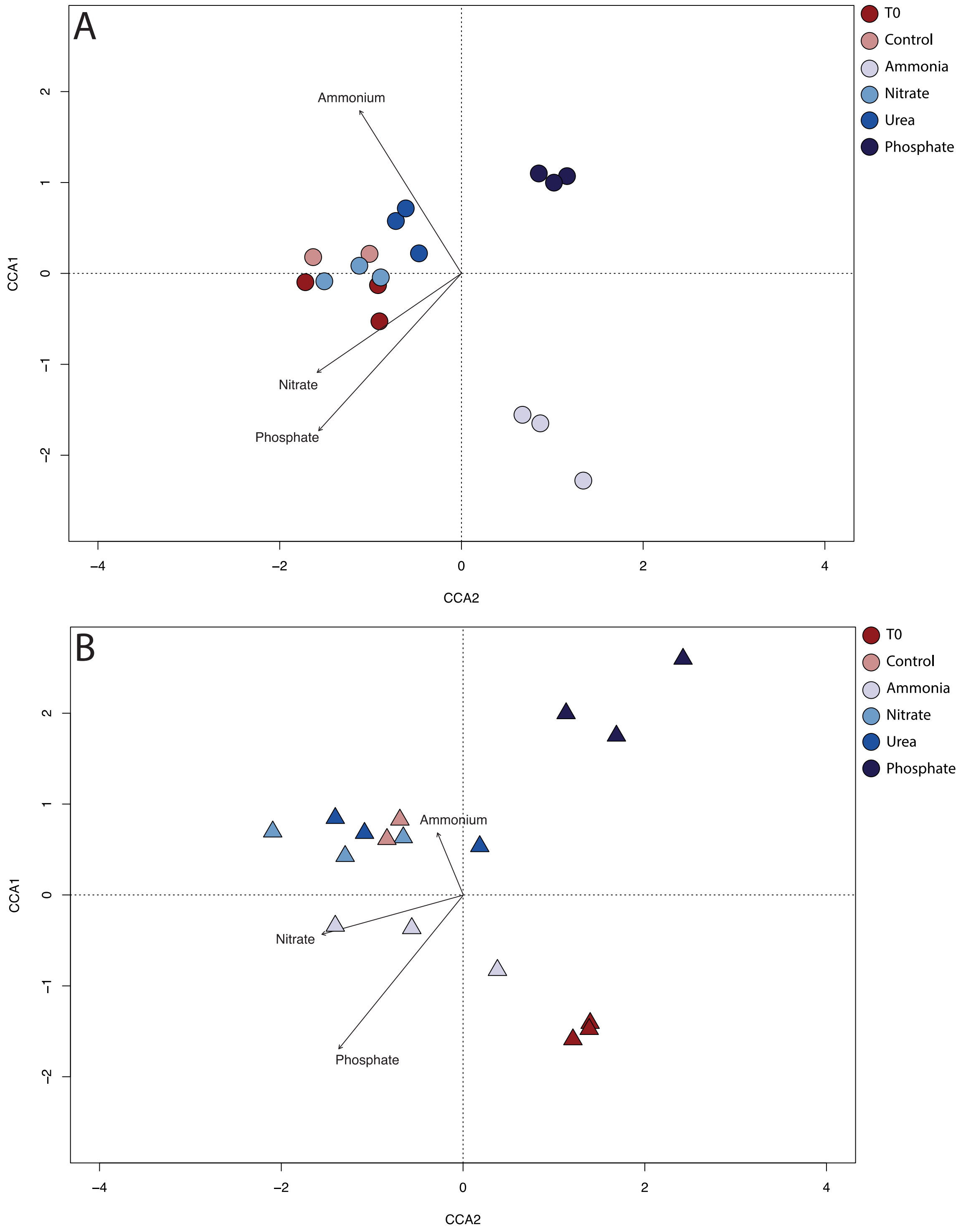

Supplemental Figure 3. Canonical correspondence analysis of *rpoB* expression. Figure 3A indicates primary producers (circles) and Figure 3B secondary producers (triangles). Nutrient treatment is distinguished by color.

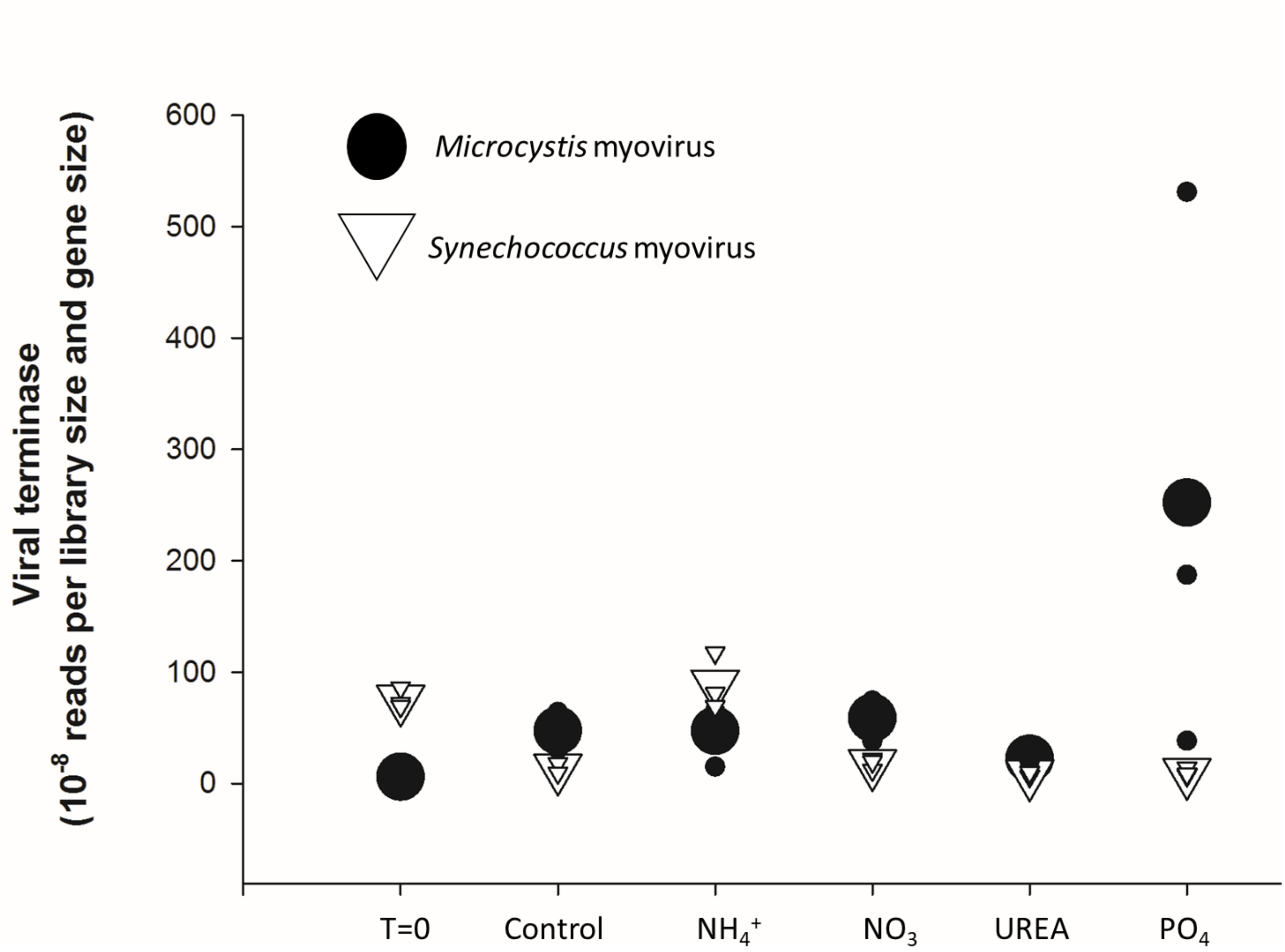

Supplemental Figure 4. Average (large markers) and individual bottle (small markers) normalized expression of a *Microcystis* phage marker (gp118 terminase, black circles) and a *Synechococcus* phage marker (gp 118 terminase, open triangles). Expression was normalized to the library size of each sample and the length of each gene.

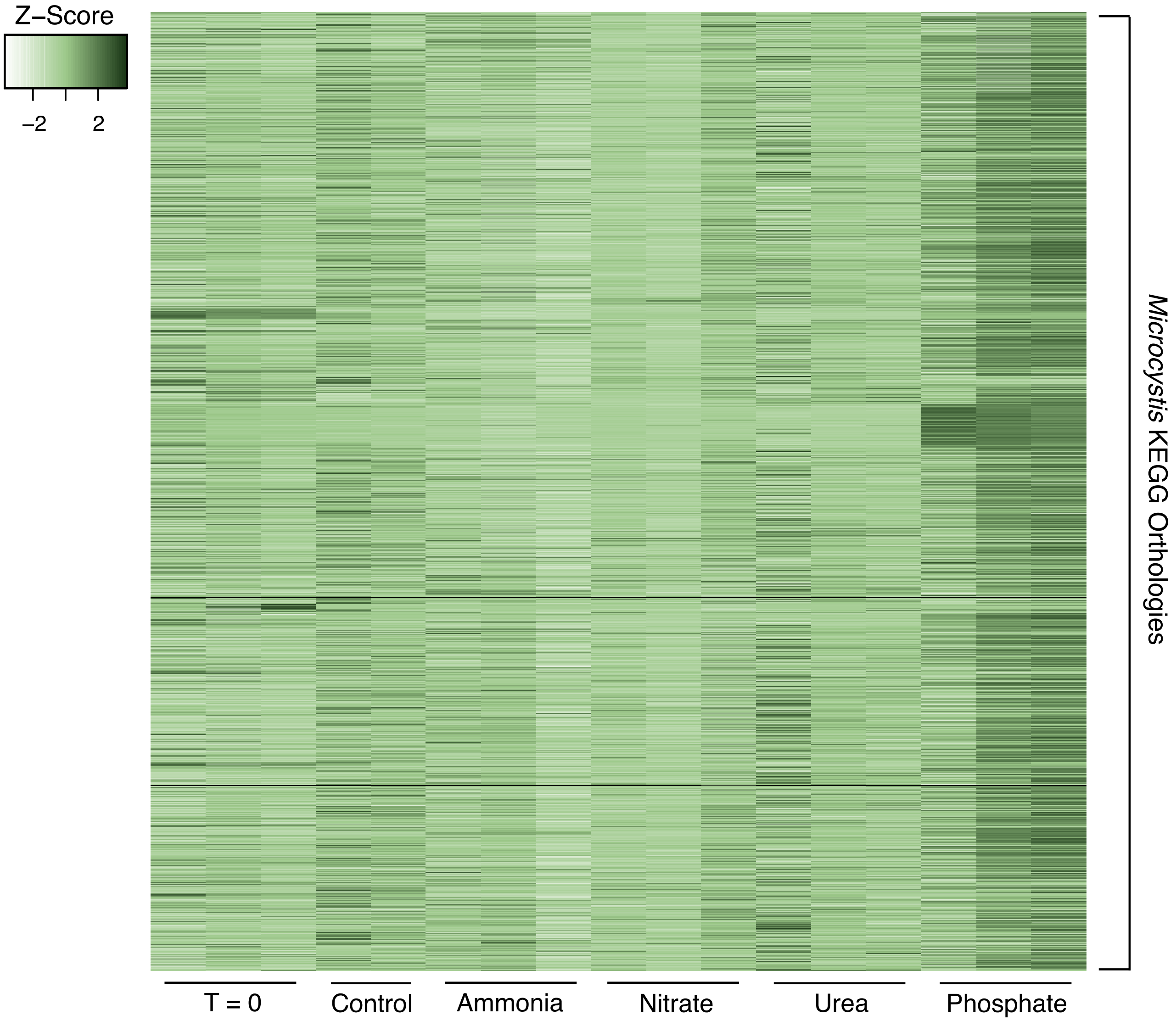

Supplemental Figure 5. Heatmap of normalized expression of all *Microcystis* spp. KEGG orthology (K number) categories. Color scale has been scaled to each K number (z-score). The expression for each annotated sequence was summed to each K number in each experimental bottle. Expression was normalized to the library size of each sample and the length of each sequence. The list of K numbers included, in the order presented here, can be found in Supplemental Data 1, Sheet "*Microcystis*_allKO_sums."
